# Ganglioside GM1 and the Central Nervous System

**DOI:** 10.3390/ijms24119558

**Published:** 2023-05-31

**Authors:** Zhongwu Guo

**Affiliations:** Department of Chemistry, University of Florida, Gainesville, FL 32611, USA; zguo@chem.ufl.edu

**Keywords:** central nervous system, glycan, glycolipid, glycosphingolipid, function, neurological disease

## Abstract

GM1 is one of the major glycosphingolipids (GSLs) on the cell surface in the central nervous system (CNS). Its expression level, distribution pattern, and lipid composition are dependent upon cell and tissue type, developmental stage, and disease state, which suggests a potentially broad spectrum of functions of GM1 in various neurological and neuropathological processes. The major focus of this review is the roles that GM1 plays in the development and activities of brains, such as cell differentiation, neuritogenesis, neuroregeneration, signal transducing, memory, and cognition, as well as the molecular basis and mechanisms for these functions. Overall, GM1 is protective for the CNS. Additionally, this review has also examined the relationships between GM1 and neurological disorders, such as Alzheimer’s disease, Parkinson’s disease, GM1 gangliosidosis, Huntington’s disease, epilepsy and seizure, amyotrophic lateral sclerosis, depression, alcohol dependence, etc., and the functional roles and therapeutic applications of GM1 in these disorders. Finally, current obstacles that hinder more in-depth investigations and understanding of GM1 and the future directions in this field are discussed.

## 1. Introduction

**Figure 1:**
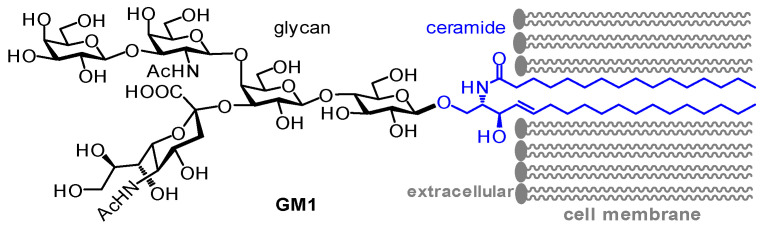
The structure of GM1 and its association with the cell membrane through insertion of the lipid tails (blue) into the membrane.

GM1 (or GM1a) is a glycosphingolipid (GSL) consisting of a pentasaccharide and a ceramide (Cer), which are coupled together by a β-glycosidic linkage (Figure 1). GM1 is one of the gangliosides, i.e., sialylated ganglio-series GSLs, since its glycan contains an *N*-acetylneuraminic acid (Neu5Ac) residue, which is the most common member of the family of sialic acids and thus is frequently referred to as the “sialic acid”. GM1 is usually anchored onto the cell surface through embedding its lipid chains into the plasma membrane bilayer, leaving the glycan as the outer leaflet (Figure 1). GM1 is one of the main GSLs in the plasma membrane of the vertebrate central nervous system (CNS) to play a key role in various functions of the CNS [1]. GM1 is also abundant in peripheral tissues closely associated with many other physiological and pathological processes [2,3,4,5]. However, this article is focused on the research progress related to the distribution and function of GM1 in the CNS, as well as its relationships with various CNS-related diseases.

## 2. GM1 Metabolism

**Figure 2:**
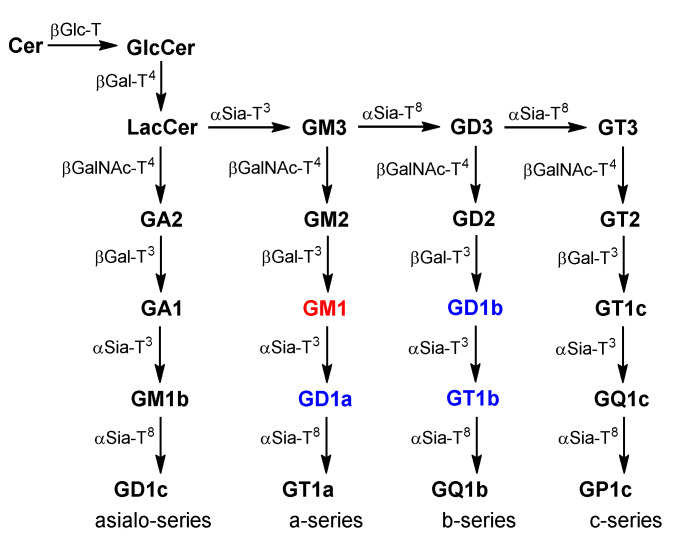
Biosynthesis of ganglio-series GSLs. The numbers in superscript indicate the glycosylation sites of GTs. GM1, GD1a, GD1b, and GT1b are the major gangliosides in the human brain.

Like other gangliosides, GM1 is biosynthesized in the endoplasmic reticulum (ER) and Golgi [6]. After Cer is generated in the ER [7], it is transported to the Golgi, where sugar residues are added sequentially by a set of membrane-associated glycosyltransferases (GTs) to furnish GM1 (Figure 2). GM3 is the common intermediate for the synthesis of all gangliosides, whereas GM1 is the immediate precursor of GD1a—another important ganglioside. Besides de novo biosynthesis, GSLs are also generated by salvage pathways [8,9]. For example, GSLs are endocytosed and degraded to monosaccharides, sphingoids, fatty acids, and simpler GSLs such as glucosylceramide (GlcCer) and galactosylceramide (GalCer), which are salvaged by the de novo biosynthetic pathway. Alternatively, after endocytosis, GSLs are directly recycled or remodeled in the Golgi before re-presentation onto the cell surface [10,11]. It has been shown that 80% of the sphingosine derived from ^3^H-labeld GM3 was metabolically incorporated by cells [12], and fluorescent *N*-[6-(7-nitrobnez-2-oxa-1,3-diazol-4-yl)aminohexanoyl]-GM1 is directly recycled by hippocampal neurons [13]. In addition, in some cells, GSLs are also remodeled by GTs [14], sialidases [15,16], and other enzymes [17] on the plasma membrane.

**Figure 3:**
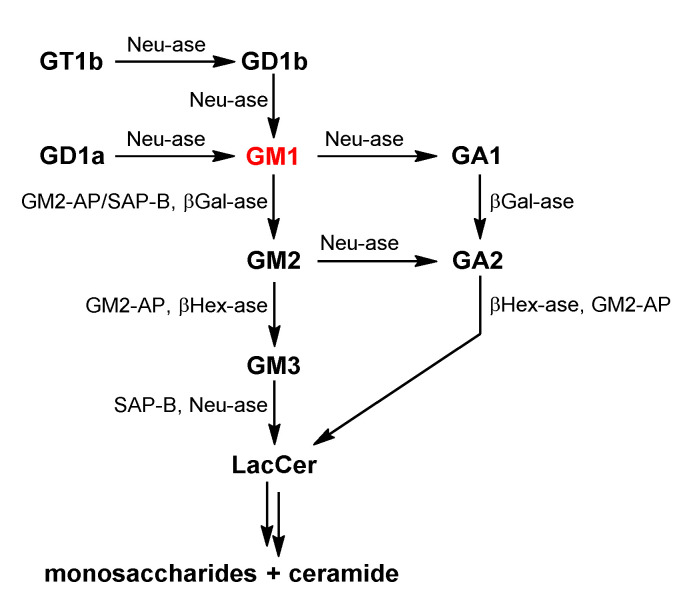
The degradation of some important gangliosides. Complex gangliosides are degraded to form GM1 on the cell surface and then translocated into the cell through endocytosis to be further hydrolyzed to free monosaccharides and Cer.

Ganglioside degradation is achieved by stepwise enzymatic hydrolysis of their glycans starting from the non-reducing end (Figure 3). Sialidase or neuraminidase (Neu-ase)-catalyzed desialylation is the first step of ganglioside degradation, occurring both on the cell surface and in the endosomes and lysosomes [6,18], where GM1 is the common intermediate. Cell surface GM1 can be endocytosed for further degradation in lysosomes [19]. The enzymes engaged in GM1 hydrolysis include Neu-ases, β-galactosidase (βGal-ase), β-hexosaminidase (βHex-ase), glucocerebrosidase (GCase), and ceramidase [20], which are typically water-soluble hydrolases. To prompt the interaction of these enzymes with membrane-associated GSLs, sphingolipid activator proteins (SAPs) are used as enzyme cofactors (Figure 3) to help extract GSLs from the membrane [6,21].

The expression levels and distribution patterns of GM1 and other gangliosides in the CNS are affected by many factors. First, the activity and location of enzymes engaged in GSL synthesis and degradation have a direct impact on GSL metabolism. Thus, development-associated changes in GSL distribution are typically the results of reprogramed enzyme expression [22]. For example, it has been shown that epigenetic activation of the GalNAc-T gene leads to GM1-induced neural stem cell (NSC) differentiation, whereas nuclear GM1 binds acetylated histones on the promoter of the GalNAc-T gene in differentiated neurons [23]. Second, forming stable clusters of various GTs is also important for the effective biosynthesis of GSLs [24,25]. Third, the levels of enzyme substrates, e.g., sugar nucleotides as glycosyl donors for GTs [26], in cells can affect glycosylation reactions and GSL synthesis. In addition, the cell membrane composition and fluidity, external stimuli, and flow rates of different GSLs through the Golgi are other factors that may also affect GSL metabolism and distribution [6,27,28]. Overall, various GTs and hydrolases form a complex and effective network in the control of GSL expression and distribution and keep them in check during delicate neurological activity. As a result, any changes in these factors, such as enzyme activity, substrate level, or environment, may break the dynamic equilibrium of GSLs and result in functional disorders.

## 3. GM1 Distribution in the Central Nervous System

It has been well established that GSLs are a major component of the cell membrane and are especially rich in the CNS. For example, >80% of the glycans in the glycocalyx of brain cell are GSLs [29,30]. Moreover, GSL expression levels and patterns are cell-, tissue-, species-, and development-specific [31]. In vertebrate brains, gangliosides are predominant, accounting for >75% of total sialic acids [29], which is 10–30 times higher than that in other tissues [32]. Overall, the expression level and structural complexity of GSLs increase with brain development but start to decrease during old age [33]. In the adult human brain, GM1, GD1a, GD1b, and GT1b are extremely enriched, representing >97% of all gangliosides [34,35]. Studies have shown that they are also the major gangliosides in the brain of many other animals [36,37,38,39,40,41,42,43]. In addition, the Cer structure, i.e., the lipid form, of GSLs is also cell-, tissue-, species-, and development-dependent [29,30].

**Figure 4:**
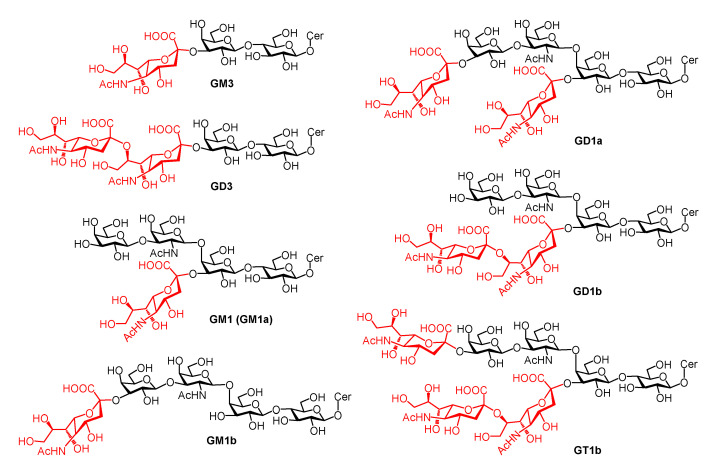
Chemical structures of the several dominant gangliosides in the vertebrate brain, (GM3, GD3, GM1, GD1a, GD1b, GT1b, GM1b) which have different numbers of sialic acid residues (in red) or have sialic acid at different locations.

Specifically, dramatic changes in the expression levels and patterns of gangliosides have been associated with the development of rat and mouse brains. Simple gangliosides (GM3 and GD3, Figure 4) are rich in the early embryonic stages, but more complex gangliosides (GM1, GD1a, GD1b and GT1b, Figure 4) are predominant in the late developmental stages of the brain [38,39,40,41,42,43]. In rats, the decrease in GM3 and GD3 and the increase in GM1, GD1a and GT1a are correlated with the increase of GalNAc-T that transforms GM3 into GM2, and at birth, GM3 and GD3 are already minor components in the rat brain. During brain maturation, the total ganglioside content can increase several fold [30]. However, the total ganglioside content of the mouse brain was almost constant from young adulthood to the beginning of senescence, despite the changes in their composition (e.g., an increase in GM1 and decrease in GD1b, GT1b and GQ1b). During senescence, however, the concentration of total gangliosides may decrease by 20% [44]. These results suggest that GSL expression is age-related.

There are also differences in the ganglioside profile in various brain regions. For example, gangliosides in the cortex, olfactory bulb, corpora quadrigemina, cerebellum, thalamic region, and medulla of the rat brain are distinct, and each region has its own unique development markers [45,46]. GQ1b and GP1 are the markers of neural cell sprouting and arborization; GD1a and GM1 are synaptogenesis and myelination markers, respectively. In the adult mouse brain, GD1b and GT1b are abundant in gray and white matter, and GM1 is dominant in white matter [42]. GD1a is the major ganglioside in the hippocampus, cortex, and caudate nucleus, but GT1b is the main species in the cerebellum, hypothalamus, and pons–medulla [47]. Similarly, significant differences in regional ganglioside distribution were also observed in newborn and adult rats. Newborns have lower ganglioside contents in their forebrain (2.5 times), cerebellum (2.0 times), and brain stem (2.0 times) as compared to the adults [48]. At the cellular level, GM1 was detected on the cell soma, while GD3 was found on non-neuronal cells but not on neuronal cells. Furthermore, a substantial quantity of gangliosides were transformed into more complex products upon neurite outgrowth and morphological changes [49].

Qualitative and quantitative studies of postmortem human brains have also revealed dramatic and steady changes in GSL profiles during neurodevelopment and neuropathogenesis [38,50,51]. For example, fetal human brains showed a stage-specific expression of GSLs [52,53]. At gestational week 10, GT1b, GD1b, and GD3 were predominant, but their ratios were reduced at week 22. GD1a and GM1 expression increased markedly between weeks 12 and 14, slowly between weeks 14 and 18, and rapidly again from week 20. A study on the human frontal lobe over a period from 10 fetal weeks to 80 years of age showed an increase in both the concentration and structural complexity of gangliosides during brain development [54]. The ganglioside content increased by roughly a factor of three from gestational week 10 to 5 years old, whereas GM1 and GD1a increased by a factor of 12–15 during the same period. The most rapid increases of GM1 and GD1a occurred around term. Other gangliosides have exhibited different developmental curves. Another study of adult human brains has demonstrated a continuous change in ganglioside composition between 20 and 70 years of age, namely a decrease in GM1 and GD1a and an increase in GD1b, GM3, and GD3 [55]. A composition of gangliosides in cortical regions further revealed region-specific changes associated with aging. In the frontal cortex, GD1a and GM1 declined with aging but no significant change was observed in the visual cortex. GD1b and GT1b in the cerebellar cortex were reduced with aging, but it was GD1a that showed a moderate decrease in hippocampus [56,57].

In vitro studies using cultured hippocampal neurons revealed that complex gangliosides such as GM1, GD1a, GD1b and GT1b were drastically increased during axonogenesis, accompanied by a decrease in GD3 and GlcCer [58]. Investigation of different types of brain cells has demonstrated the great diversity of gangliosides in their expression and distribution patterns [59]. For example, while GM1 and GM4 were found on all oligodendrocytes and most astrocytes (80%) and Schwann cells (50–70%), only GM1, not GM4, was observed on most neurons (80%) and a fraction of fibroblasts (5–10%).

The structure and distribution of Cer in GSLs are also tissue-, species-, and development-specific, and this is reflected in both the total lipids and individual GSLs [60]. While ceramides consisting of sphingosine (d18:1) and (d20:1) are predominant in mammalian brains, the constitution of sphingosine (d20:1) increases with brain development and aging [60,61]. For example, the fetal human brain contains <3% sphingosine (d20:1) [62,63,64], but it increases to roughly 50% by 30 years of age. While all (d20:1) gangliosides remarkably and progressively increase with aging, this trend is more obvious for GD1b, GT1b, GQ1b, GM1, and GD1a [64,65,66,67]. Mass spectrometry (MS) imaging of 11 regions of rat brains across their lifespan revealed an age-dependent increase in GM1 and GD1 (d20:1), especially in the early developmental stage, and a decrease in the ratio of sphingosine (d20:1)/(d18:1) for GM2 and GM3 [68]. Similar results were also observed in rats [69,70]. Interestingly, GM1 was the only ganglioside that has a higher sphingosine (d20:1) ratio in the synaptosomal fraction than in the myelin fraction. Furthermore, exogenous GM1 (d18:1) can be inserted into the cell membrane 2.43 times more than GM1 (d20:1) [71]. In regard to the fatty acyl component of Cer, palmitic (C16:0) and stearic (C18:0) groups are the most common, but the fatty acyl chain can vary from C14 to C30 [62,72]. In humans, it was found that the stearic component in GSLs decreased from 93% at birth to 78% at 98 years old, whereas C20 acyl chains increased from 3 to 9% with the most obvious changes found after 60 years of age [73]. In addition to the length of the fatty acyl group, its structure also changes [74], namely in different degrees of saturation, which can have a significant impact on the behavior and function of GSLs. As a results, brain development and neurological disorders are often accompanied by structural changes in the Cer of GSLs [75,76,77].

**Figure 5:**
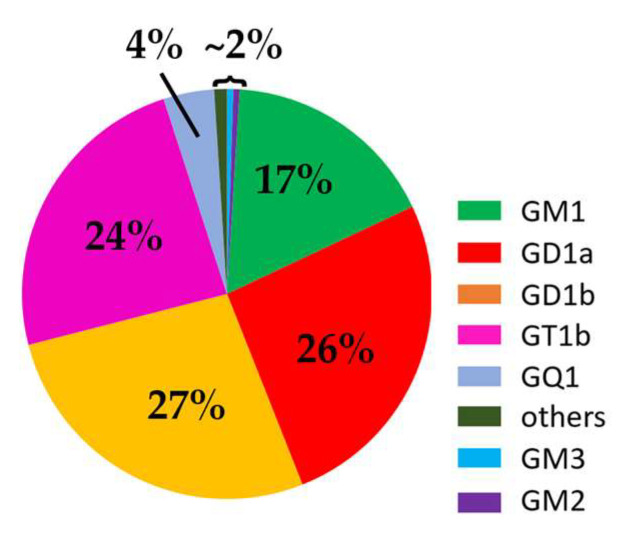
Major ganglioside species and their proportions in the adult human brain. This figure is generated using the data presented in the literature [29,35].

In summary, in the early stages of brain development, simple gangliosides such as GM3 and GD3 are expressed abundantly [66,78,79,80]. With brain maturation, a broader spectrum of more complex gangliosides is expressed, which is correlated with the increasingly sophisticated functions of the brain. In the brains of all adult mammals and birds, GM1, GD1a, GD1b, and GT1b are predominant (Figure 5) [30,35]. These four major gangliosides also exhibit tissue specificity, with GM1 and GD1a as the most abundant gangliosides in the white matter and brain nuclei and tracts, respectively [42]. The ganglioside pattern evolves continuously, and the overall trend is a gradual decrease in GM1 and GD1a and increase in GD1b, GM3, and GD3 alongside aging [81]. In the meantime, the lipid structure of GSLs is also diverse and dependent on tissues and development, which may have an impact on the organization of GSLs on cells and the fluidity or other biophysical properties of the cell membrane, thereby affecting the biological activities of GSLs. For example, it has been shown that an increase in the ratio of C20 fatty acid-containing GM1 and GD1 in the hippocampus may be associated with altered membrane fluidity and other properties and increased susceptibility of cells to degeneration during aging [60]. Similarly, lipid rafts isolated from neurons aged in vitro exhibited an increase in Cer content and a decreased level of gangliosides [82].

## 4. Biological Functions of GM1 in the Central Nervous System

GSLs are abundant, structurally diverse, and tissue-/development-dependent in the CNS, indicating that they may play a role in the regulation of neurological processes. Specific sets of gangliosides are associated with particular development stages of the brain, suggesting that they may mediate neuronal differentiation and growth [83,84]. For example, GM1 can interact with its receptors on the cell surface to activate processes such as neuronal migration, dendrite emission, neonatal cortical development, and axon growth [85,86]. Thus, GM1 is a differential marker of neurons [87]. GM1 and GQlb, but not other gangliosides, have been shown to boost neuritogenesis in neuroblastoma cells [88,89], and GM1 can accelerate neurite outgrowth from primary peripheral and central neurons [90]. GM1, GM3, and ganglioside mixtures were found to reduce the baseline rate of proliferation of Schwann cells and their response to mitogens [91].

The mechanism by which GM1 mediates neuron differentiation and growth is still not clear. However, it is generally accepted that GSLs on the cell surface may interact with specific receptors to generate signals that initiate appropriate cellular responses [83]. The interaction of GM1 with Ca^2+^ or membrane proteins is one of the potential mechanisms of GM1 to activate neuronal differentiation and development [92], which is supported by the anionic structure of the Neu5Ac residue that binds Ca^2+^ and GM1 co-localization with the Ca^2+^ pump. It was also revealed that, in the rat brain, GM1, GT1b, and GD1a could stimulate Ca^2+^/calmodulin-dependent protein kinase II (CaM-kinase II), which is independent of Ca^2+^ [93]. However, at high concentrations, GT1b, GD1a, and GM1 inhibited CaM-kinase II activity in a substrate-dependent manner. Moreover, the ability of GM1 to enhance PC12 cell fiber outgrowth involves nerve growth factor (NGF) [94], which is a key neurotrophic factor regulating neuronal proliferation, growth, maintenance, and survival [95]. Among all gangliosides that can affect the activity of NGF, GM1 is the most important. It can potentiate NGF both in vitro and in vivo [96,97] to prevent vinblastine-induced sympathectomy in newborn rats [98] and to improve the performance of cortically lesioned rats [99]. GM1 activates NGF through interacting with an NGF-associated tropomyosin receptor kinase (Trk) to promote tyrosine phosphorylation [100,101].

Many studies have revealed that GSLs can regulate signal transduction across the cell membrane via interacting with membrane proteins [102,103]. GSLs can bind proteins on the same or opposite cells and in the extracellular matrix to affect protein conformation, receptor aggregation, and receptor interaction with partners in or on the cell membrane, thereby generating binding signals. For example, GM1 interaction with TrkA was shown to lead to the dimerization and activation of this kinase and then initiation of downstream activities [100,104]. GM1 is a co-receptor of fibroblast growth factor 2 (FGF2), required for FGF2 activation [105]. GM1 also possesses neuroprotective and neurorestorative activities for several neurotransmitter systems [106] through binding neurotransmitters released at the synapse to help their presentation to high-affinity receptors [107,108,109]. GM1-mediated signaling is associated with various kinases and phosphorylation processes. For example, GM1 has been shown to trigger the phosphorylation and activation of Trk and Erk receptors [110,111] and phosphatidylinositol 3-kinase [104] and to promote neurotrophin-3 release through stimulating TrkC autophosphorylation. Changes in the endogenous GM1 density around TrkB have been shown to affect the activity of TrkB [112]. The molecular basis for the interaction between GM1 and kinases is not clear, but its glycan is important as GM1 oligosaccharide alone had a similar activity to GM1 [85,113].

One of the fundamental functions of GM1 in the CNS is to protect the nervous tissues against various toxic agents or conditions and maintain neuronal activity [114]. For example, GM1 has been shown to prevent neuronal apoptosis [115], neurodegeneration [116], and neuronal functional decay [117], as well as glutamate- and ethanol-caused neurotoxicity [118,119,120], anoxia-caused neuronal death [121], and cerebral or retinal ischemia- and trauma-induced brain and neural injuries [122,123,124,125]. GM1 has been shown to provide neural protection by a variety of mechanisms. For example, GM1 prevents apoptotic neuronal death through Trk neurotrophin receptors [126]; ketamine-induced hippocampus and cortex apoptosis and cognitive impairment through the PI3K/AKT/GSK3β pathway [127,128]; lead-caused cognitive deficit and brain damages by averting changes in superoxide dismutase and intracellular Ca^2+^ levels [129] and activating the SIRT1/CREB/BDNF pathway [130]; high altitude-caused cerebral edema by reducing oxidative stress and PI3K/AKT-Nrf2-linked inflammatory response [131]; and cognitive deficits induced by trauma [132] and MK801 [133]. GM1 has also been shown to protect the CNS via eliciting anti-inflammatory activity [134] and brain spectrin–aminophospholipid interaction [135], stabilizing plasma membrane Ca^2+^ ATPase-neuroplastin complexes [136], interacting with acetylated histones to regulate gene expressions [137], and modulating neuronal plasticity [138]. In addition, it has been found that GM1 can protect brains from neurotoxin-induced inhibition of choline uptake [139], diminish cell death caused by excessive excitatory neurotransmission [140], and reduce amyloid beta (Aβ)-related cognitive deficits and ischemia-induced brain damages by modulating Na^+^/K^+^- and Mg^2+^-ATPases [141,142,143].

GSLs play a critical role in the processes of neural cell differentiation, neurite sprouting and growth, and nerve regeneration [83,84]. However, the abilities of various GSLs to promote neuritogenesis and nerve regeneration vary dramatically. For example, GT1b and GD1b are stronger than GM1. These results suggest the potential involvement of GSL glycans in these processes [144]. The mechanisms by which GM1 influence neuritogenesis and nerve regeneration are not clear but are believed to be related to specific neurotrophic and growth factors. In rats, GM1 can associate with Trk to induce tyrosine autophosphorylation and enhance PC12 cell neurite outgrowth and neurofilament expression elicited by NGF, while NGF alone is insufficient to induce such activity [100]. It was also shown that the neurotrophic activity of GM1 was due to its activation of RET tyrosine kinase receptors via interaction with GFRα1 co-receptors in the glia cell-derived neurotrophic factor (GDNF) receptor complex [145]. These findings suggest the possibility of using GSLs to promote nerve regeneration and treat nervous injuries and neurodegenerative disorders. Indeed, GM1 has shown a positive effect on cerebrovascular [146] and Parkinson’s disease (PD) patients [147] and brain trauma in mice [132]. Recently, it was also reported that intracerebroventricular (ICV) infusion of exogenous gangliosides, such as GD3 and GM1, can promote adult neurogenesis in damaged brains [148].

GSLs also play a critical role in memory and cognition through modulating synaptic activities [32] such as neurotransmission [149], functional synapse and neuronal circuit formation and stabilization [150], etc. These actions are supported by the adoptive change of GSLs in response to brain development, sensory stimulation, CNS activity, and learning processes [151]. Exogenous GM1 was shown to induce neurotransmitter release in the cortical synaptosomes of mouse brains [152], and intraperitoneal injection of GM1 was found to improve spatial learning and memory in cognitively impaired geriatric rats [153], thereby helping improve age- and lesion-caused memory deficits [154]. GM1 was discovered to affect neurotransmission through regulating ion channel activities and related signaling, namely enhancing depolarization-induced Ca^2+^ influx into the synaptosome [152], which is also the underlying mechanism for the enhanced synaptosome release of acetylcholine (ACh) upon stimulation [155]. GM1 was also shown to affect synaptic plasticity [156], referring to the persistent changes in the strength of connections between neuronal cells [150]. Therefore, enhancing the GM1 content in the synaptic membrane of rat hippocampal slices has been proved to increase the synaptic plasticity and potentiation ability of nerves [157]. It has been further demonstrated that intraperitoneal injection and dietary supplementation of gangliosides can have a positive impact on neuroplasticity and the cognitive functions of the brain [150,158].

GM1 can influence the CNS by several other mechanisms as well. For example, it has been shown that GM1 can affect dopamine-mediated signaling through enhancing dopaminergic neuron regeneration [159,160] and high-affinity dopamine uptake in the striatal synaptosome [161], up-regulating dopamine receptors in rat brains [162], and reviving electrically stimulated dopamine release in the kainic acid-lesioned rat striata [163], thereby restoring cholinergic and dopaminergic deficits in aged rats [164], protect dopaminergic neurons against 6-OHDA (6-hydroxydopamine)-caused damages [165], and improve behavioral changes caused by irreversible blockage of dopaminergic sites [166]. GM1 is also a modulator of Ca^2+^ ion flux and equilibrium in the membrane [167,168], e.g., as a negative regulator of Ca^2+^-ATPase [169,170] or a potentiator of the nuclear Na^+^-Ca^2+^ exchanger [171,172]. GM1 can also affect the CNS through interacting with specific receptors or enzymes. For instance, GM1 was shown to enhance the efficacy and sensitivity of sensory neurons to opioid [173,174] via modulating the activity of excitatory opioid receptor [175,176] and promote leptin receptor (LepR) signaling via activating the JAK2/STAT3 pathway [177]. In addition, GM1 has been found to function by reducing the regional cerebral metabolic rate of glucose in conscious rats [178] and altering the membrane property [136].

In summary, GM1 is one of the most abundant gangliosides in the vertebrate brain and is closely related to the various functions of the CNS [179] (Table 1). GM1 expression in the CNS is dependent on development, and a decrease in GM1 content has been associated with aging, degenerated neurotransmitter activity, and age-related symptoms such as cognitive impairment [180]. In general, GM1 plays a protective role and is directly involved in cell differentiation, neuritogenesis, neuroregeneration, signaling, and neuroplasticity in the CNS. GM1 carries out its functions by an array of mechanisms. Like other GSLs, GM1 tends to cluster in the cell membrane lipid rafts, which are rich in GSLs and various receptors and signaling molecules [76,181]. However, typically, the lipid chains of GSLs are not long enough to span the entire plasma membrane. To transduce signals through the membrane, GSLs need to interact with other membrane components. As a result, lipid rafts are useful platforms for GM1 to form specific microdomains that enable GM1 interaction with molecules both inside and outside of the cell membrane. Consequently, in addition to their glycans, the Cer moieties in GM1 and other GSLs also play an important role in their biological functions [182,183,184].

## 5. GM1 in Central Nervous System-Related Diseases

### 5.1. Alzheimer’s Disease

Alzheimer’s disease (AD) is a neurodegenerative disorder that accounts for roughly 60–70% of all cases of dementia [185]. The pathogenesis of AD is not fully understood yet, but AD brains are typically accompanied by the accumulation of misfolded proteins in the form of neurofibrillary tangles. According to the amyloid cascade hypothesis, enzymatic cleavage of the transmembrane amyloid precursor protein (APP) can lead to the release of extracellular Aβ peptides that aggregate into insoluble and neurotoxic oligomers, fibrils, and plaques [186]. Furthermore, Aβ oligomers can promote tau protein hyperphosphorylation and aggregation, which is another cause of neurotoxicity. In this process, GSLs [187,188,189,190,191,192], especially GM1 [193,194,195,196,197,198,199,200,201,202,203,204], play an important role.

Many studies have shown that in AD brains, GM1 can bind monomeric Aβ to act as the seed to promote Aβ aggregation and fibrillogenesis [193,194,195,196,197,198,199,200,201,202,203,204]. After binding to GM1, Aβ performs a series of structural changes, from random coil to α-helix-rich and then β-sheet-rich conformations, depending on the concentration and the aggregation form of Aβ [205]. The β-sheet-rich aggregates are more likely to form neurotoxic oligomers and fibrils [192,206]. Other factors may also affect the process. For example, both sphingomyelin accumulation-[207] and age-dependent high-density GM1 clustering at the presynaptic neuritic terminal [208] could promote Aβ fibrillogenesis. In addition, both the glycan and the Cer of GSLs are important for their interactions with Aβ. For example, Aβ forms longer fibrils in GM1-, GD1a-, and GT1b-containing membranes than in GD1b- and GM2-containing membranes, and GM1 has exhibited the strongest activity as amyloid seeds, indicating the importance of their glycans [209,210]. Additonally, an increase in the GM1 (d18:1) to (d20:1) ratio was observed in AD patients [211]. To explain the influences of GM1 on AD and its progression, it has been suggested that a decrease in GM1 on neuronal cells activates the PI3K/Akt/mTOR pathway to inhibit Aβ fibrillogenesis [212] and, in addition, GM1 can alleviate oxidative stresses to improve memory deficits in an AD rat model [213]. In addition, the mixture of GM1 and soluble Aβ can enhance NSC proliferation [214].

However, GM1 plays a generally protective role in the CNS and is reported to diminish Aβ-associated neurotoxicity [213,215,216]. For example, GM1 can reduce Aβ-induced neurodegeneration through regulating APP proteolysis [217] and inhibiting Aβ-caused Na^+^/K^+^-ATPase inactivation [142,216] or GSK3β activation [215]. The latter signaling pathway is involved in the apoptosis and death of neural cells. In addition, GM1 can prevent sphingomyelin-triggered Aβ oligomerization [203]. The apparently contrary roles of GM1 in AD pathogenesis may be attributed to the unique properties of GM1-Aβ interaction [186]. It has been shown that Aβ deposition in the brain is region-, lipid composition-, and environment-dependent [204,205]**,** and the density and organization of GM1 in the membrane are pivotal in its ability to promote Aβ aggregation [218]. Aβ can selectively bind clustered GM1 in lipid rafts [210,219,220,221,222], whereas the physiological concentrations of GM1 are not enough to provoke Aβ oligomerization [203]. Conversely, at low densities, GM1 has been demonstrated to disrupt Aβ dimers in vivo [206] and inhibit sphingomyelin-triggered Aβ oligomerization in vitro [203].

The involvement of GM1 in AD pathogenesis suggests the potential of GM1-based therapy for AD. It has been shown that midazolam, a benzodiazepine that does not target GSLs directly but decreases GM1 expression and GM1-rich microdomains in presynaptic membranes, inhibits amyloid fibril formation in synaptosomes derived from geriatric mice [223]. In 5XFAD transgenic mice that overexpress mutant human amyloid proteins, gene manipulations to deplete GM1 and other major brain gangliosides and treatment with Neu5Ac-specific Limax flavus agglutinin result in substantial improvement to AD pathology [224]. A chimeric peptide with a strong affinity for GM1-containing raft-like membranes has shown therapeutic potential for AD [225].

### 5.2. Parkinson’s Disease

PD is another common neurodegenerative disorder, which mainly affects movements at the initial stage but, at advanced stages, other symptoms such as dementia and loss of cognition also develop [226]. PD is associated with the loss of dopaminergic neurons, but its exact cause is not clear. GSLs have been found as being involved in PD pathogenesis [227,228]. In the brain of a mouse PD model, the levels of simple GSLs were elevated while the levels of complex gangliosides were decreased, and these changes occurred in parallel with reduced GCase and increased Neu-ase activities [229]. In particular, GM1 is closely related to PD [230,231,232,233]. For example, GM1 levels were lower in residual dopaminergic neurons of PD patients than in those of the normal control, and a GM1 deficit was correlated with PD in both mice and humans [234,235,236]. In gene knockout mice, GM1 depletion led to motor disability, neurodegeneration, and other PD symptoms [237,238], which were attenuated by LIGA-20, a GM1 analogue [237]. Similarly, increasing the levels of GM1 was shown to provide protection in both chemically and α-synuclein induced PD animal models [239,240,241,242]. Thus, GM1 plays a mainly protective role against PD-related damages such as dysfunction of excitatory amino acid neurotransmitters, disorder in calcium homeostasis, abnormal metabolism of oxygen free radicals, abnormal trace elements distribution and/or deposition, and excessive apoptosis [243].

GM1 performs its protective role against PD by regulating α-synuclein folding and aggregation [239] and stimulating autophagy and α-synuclein clearance [240]. GM1 has been shown to specifically interact with α-synuclein, recruit it to the caveolae and lipid rafts, and inhibit its fibrillation [244]. GM1-containing vesicles can interact with α-synuclein to decrease its fibril formation [245]. In addition, GM1 has been shown to inhibit neuroinflammatory responses by decreasing IL-1β expression and increasing IL-1Ra expression. Due to its protective effect, GM1 may be used to treat PD. Indeed, GM1 has been shown to relieve PD-related symptoms and promote patient recovery [246,247,248]. A 5-year open study has revealed that the long-term use of GM1 is safe and may provide clinical benefits in PD patients [249].

### 5.3. GM1 Gangliosidosis

GM1 gangliosidosis is a lysosomal storage disease (LSD) caused by mutations of the *GLB1* gene, which encodes βGal-ase—the enzyme hydrolyzing GM1 into GM2 (Figure 3). The depletion or attenuation of βGal-ase activity leads to a buildup of GM1 and related molecular species [250,251]. GM1 accumulation in the CNS causes progressive neurodegeneration and neurological dysfunction. Based on onset time and severity of symptoms, GM1 gangliosidosis is divided into three clinical types: infantile (type I), juvenile (type II), and adult or chronic (type III) [252]. The symptom onset time also reflects the degree of residual βGal-ase activity remaining in the patient. An increase of GM1 in the patients’ cerebrospinal fluid was also observed; thus GM1 can be a biomarker for clinical diagnosis of the disease and assessment of therapies [253,254].

How GM1 accumulation induces neurodegeneration is not fully understood [251]. However, it has been observed that excess GM1 accumulation can cause CNS myelin deficiency [255,256,257], lysosomal swelling, and cellular dysfunction [258]. In addition, GM1 accumulation results in immune activation and inflammation [259] in the CNS and deplete Ca^2+^ in the ER to cause neuronal apoptosis [260] and reduce Ca^2+^ flux in synaptosomes, thereby impairing Ca^2+^ homeostasis [168,261,262], neurotransmitter uptake, and membrane fluidity [263]. In a mouse model of GM1 gangliosidosis, enhanced autophagy and mitochondrial dysfunction have been correlated with disease progression [264]. GM1 may also act by enhancing NGF-induced Trk receptor autophosphorylation and dimerization to elevate neurotrophic activity [113,265].

Currently, there has been no cure for GM1 gangliosidosis, and thus all treatments are symptomatic. However, significant progress has been made in several directions. For example, enzyme replacement therapies (ERT) based on ICV injection or efficient enzyme delivery systems such as “Trojan horse” proteins and nanoparticles to address the blood brain barrier (BBB) problem have been explored [266,267,268,269,270]. Chaperon-mediated therapies (CMT) employing small molecule chaperons, such as Gal [271], iminosugars and valienamine derivatives [272,273,274,275,276,277,278,279,280], have been utilized to treat diseases caused by unstable or misfolded mutant enzymes [281,282,283,284]. Some of the therapies result in significant increases in βGal-ase activity [284] and decreases in the GM1 content of neonatal brains [279]. Substrate reduction therapy (SRT) using Miglustat, an inhibitor of glucosylceramide synthase, was shown to reduce GM1 and inflammation and improve function of the CNS in animals [279,285] and reverse disease progression in patients [286]. However, SRT relies on residual βGal-ase activities to degrade the GM1 already accumulated in the CNS. Gene therapy is a promising method for GM1 gangliosidosis despite the challenge of achieving global distribution of genomes or active enzymes in the CNS [287]. In a mouse model, intravenous (IV) or adeno-associated virus (AAV)-assisted delivery of genes was shown to completely repair enzyme deficiency and lysosomal storage in the brain [288,289] and improve animal survival [290,291]. The distribution of intraparenchymally injected AAV vectors in the brain and system is believed to be achieved through diffusion, axonal transportation from neurons at the injection site, and cerebrospinal fluid (CSF) flow in the Virchow-Robin perivascular space [292]. AAV-mediated delivery of *GLB1* in a feline model has provided additional support for its therapeutic efficacy in humans [293]. However, intracranial injection suffers from the drawback of invasiveness. Recently, it has been shown in an adult mouse model that IV injection of an AAV9-*GLB1* vector, which could penetrate the BBB and target the brain [294], resulted in CNS expression of βGal-ase to significantly ameliorate the disease symptoms and extend the animals’ lifespan [291]. IV injection of the AAV9-*GLB1* vector in a cat model of GM1 gangliosidosis gave similar results. These encouraging results support the potential clinical benefits of gene therapies to treat GM1 gangliosidosis [295]. Consequently, gene therapies based on cisterna magna delivery of LYS-GM101 and PBGM01 and IV delivery of AAV9-GLB1 have been approved for clinical trials for type I and type II GM1 gangliosidosis [250,251].

### 5.4. Huntington’s Disease

Huntington’s disease (HD) is a neurodegenerative disorder characterized by progressive motor, cognitive, and psychiatric dysfunctions [296]. It is caused by mutations in the *HTT* gene encoding the Huntington protein (HTT). These mutations result in HTT misfolding and aggregation, and subsequently neuronal dysfunction and death in the striatum and cortex [297,298,299,300]. It has been revealed that HD is associated with abnormal GSL expression. For example, a decrease in striatal gangliosides, most significantly GM1, was observed in both rat models and HD patient tissues [301,302,303], but in the cerebellum, a region largely unaffected by HD, the GM1 level was elevated [304]. As discussed earlier, GM1 plays an overall protective role in the CNS, and a decrease in GM1 renders neuronal cells more susceptible to injuries and growth factor deprivation involved in the activation of the neuronal prosurvival PI3K/AKT pathway [303]. Meanwhile, GM1 was able to reduce protein aggregation and toxicity by promoting mutant HTT phosphorylation [305,306] and accelerate mutant HTT clearance from the brain [307]. Furthermore, it has been revealed that mutant HTT shows decreased insertion and oligomerization into GM1-modified membranes made from brain lipid extracts [308].

Currently, there is no cure for HD; thus all mainstream treatments are symptomatic [298,299]. GM1 is a promising prospect for HD therapy. In HD mice, intraventricular infusion of exogenous GM1 has shown many therapeutic benefits, including reversal of motor, cognitive, and psychiatric symptoms; slowdown of myelin atrophy and neurodegeneration; improvement of neurotransmitter levels [305,307]; a decrease in mutant HTT; and recoveries at the molecular, cellular, and behavioral levels.

### 5.5. Epilepsy and Seizure

Epilepsy is a neurological disorder characterized by seizures caused by aberrant neuronal activities in the brain. Severe epilepsy is often accompanied by abnormal GSL expression [29]. For example, decreased levels of the major gangliosides, including GM1, in the hippocampus and particularly GM1 and GD1a in the CSF were observed in a pilocarpine-induced mouse model of epilepsy [309] and the infantile epilepsy disease West syndrome [310]. Defects in the GM3 synthase gene in some Amish families, which result in the loss of all gangliosides, including GM1, cause serious infantile epileptic disorders [311]. Anti-GM1 antibodies were found to cause seizures and were utilized to generate epilepsy models [312]. Conversely, GM1 administration was shown to protect animals from glutaric acid-induced seizures [313].

The protective function of GM1 against seizures is believed to be associated with its ability to modulate membrane receptors and ion channels. GluR2-AMPAR is a receptor by which gangliosides affect neuronal cells [314]. It has been demonstrated that AMPARs containing GM1-bound GluR2 are functionally segregated from GT1b-bound AMPAR-trafficking complexes and disrupting the biosynthesis and balance of gangliosides results in a reduced synaptic expression of GluR2-contianing AMPARs and, thereby, intellectual deficits and seizure susceptibility in mice and humans.

### 5.6. Amyotrophic Lateral Sclerosis

Amyotrophic lateral sclerosis (ALS) is a fatal neurodegenerative disease characterized by progressive loss of motor neurons in the brain and spinal cord [315]. It is associated with significantly elevated levels of GM1 and several other simple GSLs in the spinal cord of ALS patients [316] and ALS mouse models [316,317]. In addition, GM1 levels in the CSF are correlated with disease severity [318]. GSLs may contribute to ALS via interaction with NGF and protein kinases [319,320]. Another theory is that ALS may involve autoimmunity because antibodies against GM1 [321,322] and other gangliosides [323] have been detected in the sera and CSF of most ALS patients. However, the autoimmune theory is controversial, as some studies did not observe clear association of the ALS phenotype with elevated anti-ganglioside antibodies [324].

### 5.7. Ischemic Stroke

Stroke is one of the leading causes of human death and is characterized by severe neurological deficits in long-term survivors [325]. A substantial reduction in total gangliosides, including GM1, is associated with ischemic stroke in neonatal patients who died of hypoxia [326]. Similar results were observed in animals subjected to hypoxia–ischemia [327] or middle cerebral artery occlusion (MCAO) [328]. Meanwhile, the decrease in brain gangliosides correlated with the severity of brain damage. In addition, the ratio of GM1 (d20:1) lipid form, which is neurotoxic, was also significantly elevated [328,329]. However, changes in GM1 levels vary in different regions of the brain. An increase of GM1 was observed in adult rats’ cerebral cortices that were damaged by ischemic infarct, which may be an autologous mechanism developed by adults to protect the brain from ischemic damages [330].

In a rat model of cerebral ischemia, GM1 was revealed to downregulate the *N*-methyl-D-aspartate (NMDA) receptor that regulates excitotoxicity, thereby ameliorating aberrant excitatory amino acid neurotransmitter and mitochondrial Ca^2+^ release and reuptake and reducing reactive oxygen species and associated oxidative stress in the cerebral cortex and hippocampus of the injured regions [331,332]. For example, GM1 has been found to protect the brain against ischemic injuries via preventing hypoxia-induced changes in neurotransmitters, such as choline, dopamine, and glutamate [333,334]. In a rat model of hypoxia induced by high altitudes, GM1 was found to activate the PI3K/AKT-Nrf2 signaling pathway, resulting in the suppression of oxidative stress and inflammation [131]. GM1 can also ameliorate cerebral ischemic injury via inhibition of lipid peroxidation, which results in up-regulated expression of tyrosine hydroxylase and, hence, restoration of the tyrosine/dopa pathway [122]. In a rat model of stroke with diabetes, GM1 was proved to inhibit proline-directed kinase ERK1/2 phosphorylation [335] and ER stress-induced neuronal cell apoptosis [336]. In addition, GM1 was able to reverse ischemia-induced changes in Na^+^/K^+^- and Mg^2+^-ATPase and plasma membrane disorganization, thereby leading to the reduction of ischemic injury and cell death [143,337].

The above results suggest the potential benefit of increasing brain GM1 levels for managing stroke- and hypoxia-induced tissue damages and post-stroke patients. Indeed, GM1 administration has been shown to reduce neuronal damage, functional deficit, and autophagy caused by ischemia or stroke [338,339,340,341,342,343], protect the brain from ischemic injury [123,124], and improve therapeutic outcomes and recovery. However, the protective effect of GM1 was time-dependent; thus early administration provided better protection [331]. Oral administration of LIGA20, a GM1 analogue could reduce infarct volume and behavioral impairment after focal cerebral ischemia [344]. Finally, gangliosides were found to reduce overall mortality after global ischemia [345].

### 5.8. Depression and Anxiety

Depression and anxiety are correlated with the dysregulation and/or malfunction of neurotransmitters and their receptors [346]. Gangliosides are co-receptors for some neurotransmitters that regulate their interaction with membrane receptors and their functions and, hence, are associated with depression and anxiety. It has been shown that serotonin can bind to gangliosides in the cell membrane and, in the process, the interaction of negatively charged Neu5Ac in GM1 with divalent cations, such as Ca^2+^ and Mg^2+^, plays a pivotal role [107,347]. Exogenous GM1 was also found to increase the affinity of serotonin for its receptor by a factor of ten to markedly elevate the activity of serotonin and the level of cyclic AMP [348]. Molecular dynamic simulation further suggested that the glycan of GM1 could interact with the extracellular loop of serotonin receptors to drive a conformational change that favors ligand binding [349]. GM1 showed a similar impact on dopamine and other membrane-binding neurotransmitters. GM1 was found to increase the affinity of neuronal dopamine transporters in the membranes of rat striatal synaptosomes [161]. Treatment of animals with GM1 reduced ethanol-induced locomotion decrement, anxiety, and other intoxicating effects [350,351]. In addition, GM1 treatment was proved to reverse defeat stress-induced reduction of social interaction in mice through activating the neurotrophic factor signaling cascade [352]. GM1 can form a charge-based vestibule in front of the postsynaptic membrane to attract neurotransmitters, such as histamine and dopamine, to foster their membrane association [353].

### 5.9. Autism

Autism is a neurodevelopmental disorder characterized by a wide spectrum of deficiencies in social skills and communication. It is correlated with an increase in all major brain gangliosides, including GM1, in the patients’ CSF, which may affect synaptic transmission and activities [354]. Elevated serum levels of GM1 [355] and anti-GM1 antibodies [356] are also observed in autistic children, and the antibody levels have a positive correlation with autistic severity. However, the relationship of anti-GM1 antibodies to autism is ambiguous. Some studies suggest at least partial involvement of anti-GM1 antibodies and autoimmune neuropsychiatry in autism [356], whereas other studies suggest a lack of direct relationship between them [357].

### 5.10. Alcohol Dependence

Alcohol dependence is characterized by patients’ physical and psychological reliance on ethanol, a neurotoxin that affects embryogenesis, cell migration and differentiation, and synaptogenesis [358]. Alcohol dependence is related to GSL because alcohol has an impact on the composition, orientation, and conformation of cell surface GSLs and thus the structure and function of cell membrane [359]. Oral (chronic) consumption of alcohol was found to induce a significant change in ganglioside patterns and content in the rat brain. The most significant impacts were observed with hypothalamus GD1b and GT1b, thalamus GM1 and GD1a, and hippocampus GM1, GD1b, and GT1b [360,361]. Both intraperitoneal injection and oral administration of alcohol could lower the level of GM1 in the serum of mice [362].

Accordingly, GM1 is expected to exhibit a protective effect against alcohol intoxication. Indeed, it has been discovered that daily treatment of animals with GM1 can prevent the development of physical dependence on ethanol and reduce ethanol withdrawal syndrome [363]. Pre-incubation of rat cerebellar granule neurons with GM1 or LIGA20 could reduce alcohol-induced cell apoptosis and activation of the DNA cleavage enzyme cytosolic caspase-3 [364]. Similar results were observed in developing mouse brains [365]. In particular, GM1 can attenuate ethanol-induced neurotoxicity in septal and hippocampal neurons from fetal rats and dorsal root ganglion neurons from embryonic chicks [120]. GM1 treatment could remarkably reduce the retardation of cortical cell development and arborization due to in utero ethanol exposure [358]. Pretreatment of pregnant rats with GM1 could reduce fetal alcohol effects in rat pups [366] and the development of alcohol tolerance [367]. Intraperitoneal injection of GM1 was proved to reduce the extent and duration of alcohol-induced ataxia and prevent tremors and anxiety-like behaviors associated with alcohol withdrawal [351].

The protective effect of GM1 against alcohol-induced neurotoxicity is mainly due to its ability to inhibit changes in the cell membrane induced by alcohol, thereby minimizing its impact on brain maturation and behavioral alteration [366,368]. GM1 was shown to reduce alcohol-induced activation of phospholipase A2 (PLA2), an enzyme that regulates phospholipid metabolism, in synaptosomes [369]. GM1 was also found to mitigate alcohol-induced changes in unsaturated fatty acid incorporation into cerebral phospholipids in mice, which is a mechanism to repair alcohol-caused damages [370].

### 5.11. Bacterial Infections

GSLs also play a key role in the neurotoxicity of some bacterial infections. Typically, these bacteria need to interact with GSLs on the host cell surface, either directly or through their toxins, to cause neurological damage. *Vibrio cholerae* and *Escherichia coli* are such bacteria and use GM1 as the binding receptor [371]. Cholera toxin from *V. cholerae* consists of an A subunit and five identical B subunits, which bind to GM1 on the host cell membrane to help the entry into cells [372,373]. The heat-labile enterotoxin of *E. coli* is similar to cholera toxin both structurally and functionally [371]. It was found that treating cholera toxin-resistant cells with GM1 can sensitize the cell to this toxin [371]. Studies on different lipid forms of GM1 have proved the importance of its lipid structure in toxin binding and trafficking [374]. These toxins bind only to GM1 carrying unsaturated lipids for their sorting to the Golgi and ER. Moreover, it is mainly the B subunit that interacts with GM1 in the lipid rafts, leading to the modulation of the architecture and dynamics of the membrane and then toxin uptake in the cell via endocytosis [375]. Thus, GM1 on the host cell not only functions as a binding site for the toxin but also facilitates its transmembrane movement [376]. It has been further shown that the cholera toxin B subunit can stimulate Ca^2+^ influx gated by interaction with GM1 and neuritogenesis [377], although there are also contradictory reports [378]. Tetanus toxin (TeNT) secreted by *Clostridium tetani* is another toxin that has a high affinity for GM1 and other gangliosides [371,379]. However, gangliosides with a disialyl moiety attached to the inner Gal residue, such as GD1b, appear to have the highest affinity for this toxin [380].

Based on the above findings, it is expected that reagents that can interrupt GSL–toxin interactions may be useful for the treatment of related infectious diseases. Indeed, it has been demonstrated that synthetic sialooligosaccharides mimicking the glycan of GM1 can efficiently inhibit cholera toxin binding to treat cholera-related gastrointestinal symptoms [381]. Moreover, the ability of these toxins to specifically bind GSLs and target the ER-associated protein degradation (ERAD) mechanism has provided the opportunity to develop new therapeutics for cancer and genetic diseases involving misfolded proteins. To this end, GM1-binding cholera toxin should be especially useful [382].

In summary, many neurological diseases are accompanied by changes in the expression level and lipid form of GM1 (Table 2). The correlation of such changes with and their impacts on various disorders are different. The functions and pathological mechanisms of GM1 are relatively well defined in some diseases but ambiguous in others. Nevertheless, the observation that GM1 is associated with various neurological disorders has provided the opportunity to develop new diagnostic and therapeutic strategies.

## 6. Concluding Remarks

GM1 is one of the most abundant GSLs in the vertebrate animal brain, and its expression is cell-, tissue-, species-, and development-dependent. Like other complex GSLs, the expression level of GM1 in the brain increases from the embryonic stage to adulthood and starts to decline in old age, which coincides with the development of the brain, its functions, and then progressive neurodegeneration during senescence. Therefore, GM1 is associated with a broad spectrum of biological processes and activities of the CNS, such as neuronal differentiation and growth, neuritogenesis, neuroregeneration, cell signal transduction, memory, and cognition. GM1 performs its functions via a variety of mechanisms, from cis and trans interactions with specific receptors and modulation of ion channels and pumps on the cell surface to affecting neurotransmitters and cell membrane properties, namely, the formation and stabilization of lipid rafts. In this process, both the glycan and lipid of GM1 are important. Due to the diverse functions of GM1 in the CNS, changes in its expression level are often accompanied with neurological disorders [383].

Overall, GM1 is protective for the CNS and promotes functional recovery of the brain from various diseases and pathological damages [125,384]. Therefore, elevating the CNS level of GM1 should be beneficial to most neurological disorders discussed above. In addition, GM1 has also been found to protect the brain from damages induced by neurotoxic chemicals [385,386,387,388,389,390,391,392,393,394], X-ray radiation [395,396], stimuli [397], and brain injuries [398,399,400,401], and to improve the therapeutic outcome of experimental allergic neuritis [402]. The functions of GM1 in protecting the brain from neurological disorders are generally related to its ability to interact with the receptors involved in specific signaling pathways, restore the activity of neurotransmitters, promote neuronal cell regeneration, and sustain the structural integrity and functions of the cell membrane [403]. For example, GM1 was found to enhance choline acetyltransferase activity and repair cholinergic deficits in aged and vincristine-treated rats [404,405,406,407], restore the dopamine pathway and reverse dopamine receptor supersensitivity in mitochondrial permeability transition pore (MPTP)-treated mice and monkeys [408,409,410], and ease NMDA receptor-modulated release of excitatory amino acids [411]. Similarly, the GM1 analogue LIGA20 was proved to increase the striatal dopamine level in MPTP-treated mice [412]. Furthermore, GM1 was discovered to stimulate the collateral sprouting of dopaminergic axons in unilateral hemitransection-damaged rat brain [413], prevent GM2-caused inhibition on axonal regeneration [132,414], promote the recovery of dopaminergic neurons in MPTP-treated mice and monkeys [415], rescue 1-methyl-4-phenylpyridinium (MPP)-damaged dopaminergic neurons [416], and eliminate neuroleptic-induced sensorimotor deficits [417].

The correlation of changes in the brain level of GM1 with various neurological disorders renders GM1 a promising target for new therapy development. Some of the strategies include modulating the CNS level of GM1 through direct supplement of GM1, managing endogenous GM1 by genetic methods or manipulation of related enzymes or biosynthetic precursors, and the combination of different strategies. As discussed above, some of these therapies have demonstrated significant progress. However, for all the strategies, a major issue is how to deliver the therapeutic agents to the CNS through the BBB.

Despite the great progress presented above, there are still unanswered questions concerning the functions of GM1 and its roles in neurological diseases. Our understanding of the functions of GM1 is far from comprehensive, and there is still a lack of large-scale and systematic studies of GM1 in relation to neurological disorders that employ human brain samples, but this type of study is necessary to obtain more reliable data. To gain more insights into the functions and functional mechanisms of GM1 in the CNS and its related diseases, there are several issues that need to be addressed. First, it is necessary to have access to human brain tissues at different developmental and pathological stages on a large scale. Second, GM1 is present in the lipid rafts of cell membrane, which has a complex and dynamic environment, and interacts with diverse biomolecules. To distinguish and examine the interactions of GM1 with various molecules on the cell is a significant challenge. This may be addressed by developing differently labeled GM1 probes. Third, probes available for the study of GM1 and related biology are limited. To address this problem, more effective synthetic methods are desired to provide functionalized GM1 derivatives that are enabled by modern analytical technologies. Fourth, a high-throughput method is required for large-scale and comprehensive analysis and profiling of brain GM1, including its complex lipid forms. Nevertheless, we believe that with the development of new analytical technologies and synthetic methods for GM1 and its probes, our understanding of the functions and functional mechanisms of GM1 and its relationships with neurological disorders will improve, which will help the design of new strategies for the diagnosis, prevention, and therapy of various neurological diseases.

## Figures and Tables

**Table 1:** Major functions of GM1 in the central nervous system.

CNS Functions That Involve GM1	References
GM1 is a differential marker of neurons GM1 is involved in neuronal differentiation and development and nerve regeneration GM1 promotes neuritogenesis GM1 accelerates neurite outgrowth GM1 regulates signal transduction GM1 inhibits neuronal apoptosis GM1 prevents neuronal functional decay GM1 is protective against neurodegeneration, neurotoxicity, and neuronal injury and death caused by various factors GM1 is involved in memory and cognition via modulating the synaptic activities	[87] [83,84,92] [88,89] [90] [102,103] [115] [117] [116,118,119,120,121,122,123,124,125] [32,149,150]

**Table 2:** GM1 in CNS-related neurological disorders.

Diseases	Correlation of GM1 with the Disease	References
Alzheimer’s disease	Age-dependent clustering of GM1 at the presynaptic neuritic terminal Increased GM1 (d18:1) to (d20:1) ratio At low density, GM1 disrupts Aβ dimers and inhibits sphingomyelin-triggered Aβ oligomerization Clustered GM1 in the lipid rafts binds to Aβ selectively to promote Aβ aggregation and fibrillogenesis	[208] [211] [203,206] [210,219,220,221,222]
Parkinson’s disease	GM1 deficit is observed in PD patients and animal models GM1 can stimulate autophagy and α-synuclein clearance and modulate α-synuclein folding and aggregation	[234,235,236] [239,240]
GM1 gangliosidosis	GM1 accumulation is observed in the central nervous system, including the cerebrospinal fluid	[250,251,253,254]
Huntington’s disease	Decreased GM1 in HD patients and animal models GM1 accelerates mutant HTT clearance and reduces mutant HTT aggregation and toxicity	[301,302,303] [305,306,307]
Epilepsy, seizure	Decreased GM1 Anti-GM1 antibody can cause seizure	[310] [312]
Amyotrophic lateral sclerosis	Elevated GM1 in the spinal cord	[316]
Ischemic stroke	Decreased GM1 Elevated level of GM1 (d20:1) lipid form	[326] [328,329]
Depression, anxiety	GM1 is a co-receptor of serotonin, dopamine and other membrane-binding neurotransmitters GM1 interacts with serotonin receptor to enhance ligand binding	[107,161,347] [349]
Autism	Elevated GM1 Anti-GM1 antibodies are observed	[354,355,356]
Alcohol dependence	Decreased GM1 in animal models GM1 inhibits alcohol-induced changes in the cell membrane	[362] [366,368]
Bacterial infections	GM1 is the binding receptor for some bacterial toxins The lipid form of GM1 is important for toxin binding and trafficking	[371,372,373] [374]

## Data Availability

Not applicable.

## Acronyms

AAV: adeno-associated virus; Aβ: amyloid beta; Ach: acetylcholine; AD: Alzheimer’s disease; ALS: amyotrophic lateral sclerosis; APP: amyloid precursor protein; βGal-ase: β-galactosidase; βHex-ase: β-hexosaminidase; BBB: blood brain barrier; CaM-kinase: Ca^2+^/calmodulin-dependent protein kinase; Cer: ceramide; CMT: chaperon-mediated therapies; CNS: central nervous system; CSF: cerebrospinal fluid; ER: endoplasmic reticulum; ERAD: ER-associated protein degradation; ERT: enzyme replacement therapies; GDNF: glia cell-derived neurotrophic factor; FGF2: fibroblast growth factor 2; GalCer: galactosylceramide; GCase: glucocerebrosidase; GlcCer: glucosylceramide; GSL: glycosphingolipid; GT: glycosyltransferase; HD: Huntington’s disease; HTT: huntingtin protein; IV: intravenous; ICV: intracerebroventricular; LepR: leptin receptor; LSD: lysosomal storage disease; MCAO: middle cerebral artery occlusion; MPP: 1-methyl-4-phenylpyridinium; MPTP: mitochondrial permeability transition pore; MS: mass spectrometry; Neu5Ac: *N*-acetylneuraminic acid (or sialic acid); NGF: nerve growth factor; NMDA: *N*-methyl-D-aspartate; NSC: neural stem cell; 6-OHDA: 6-hydroxydopamine; PD: Parkinson’s disease; PLA2: phospholipase A2; SAP: sphingolipid activator protein; SRT: substrate reduction therapy; TeNT: Tetanus toxin; Trk: tropomyosin-receptor kinase.
